# An overview of the viral haemorrhagic fevers for the primary care doctor

**DOI:** 10.4102/safp.v62i1.5116

**Published:** 2020-06-26

**Authors:** Indiran Govender, Olga Maphasha, Selvandran Rangiah

**Affiliations:** 1 Department of Family Medicine, Kalafong Hospital, University of Pretoria, Pretoria, South Africa; 2 Department of Family Medicine, University of KwaZulu-Natal, Durban, South Africa

**Keywords:** Ebola virus disease, travel history, Lassa fever, Lujo virus, Crimean-Congo haemorrhagic fever, Rift Valley fever

## Abstract

The viral haemorrhagic fevers are infectious diseases that often cause life-threatening illnesses. These diseases are common in the tropical areas of the world, and travel history to an endemic area together with recognising signs and symptoms is essential to aid diagnosis. Treatment is often supportive, and infection control measures need to be instituted early at the point of entry. In this article, we will provide an approach to a patient with viral haemorrhagic fevers in a primary healthcare setting.

## Introduction

‘Viral haemorrhagic fevers’ (VHFs) is a general term that refers to a group of illnesses caused by a number of distinct families of viruses.^1^ Multiple organ systems are affected by this virus, with significant damage to the vascular system.^2^ Walls of small vessels become more permeable, and clotting mechanisms are affected. Impaired autoregulation results in symptoms that are often accompanied by haemorrhage, which may be mild to life-threatening.^2^

Fever and haemorrhage can be caused by many conditions.^3^ It is therefore important to recognise VHFs at an early stage to initiate appropriate and correct treatment. In addition, fatigue, dizziness, muscle and joint pain as well as generalised weakness occur early.

The VHFs have a propensity for person-to-person spread and high mortality rates, necessitating special infection control measures when managing suspected or confirmed cases.^3,4^ However, not all the viruses associated with VHFs are lethal or spread easily amongst humans. Some less pathogenic viruses are managed as severe diseases in countries where they are usually absent in order to prevent introduction of these viruses. Large nosocomial outbreaks have included Crimean-Congo haemorrhagic fever, Ebola virus disease, Marburg virus disease and Lassa fever.^5^

**FIGURE 1:**
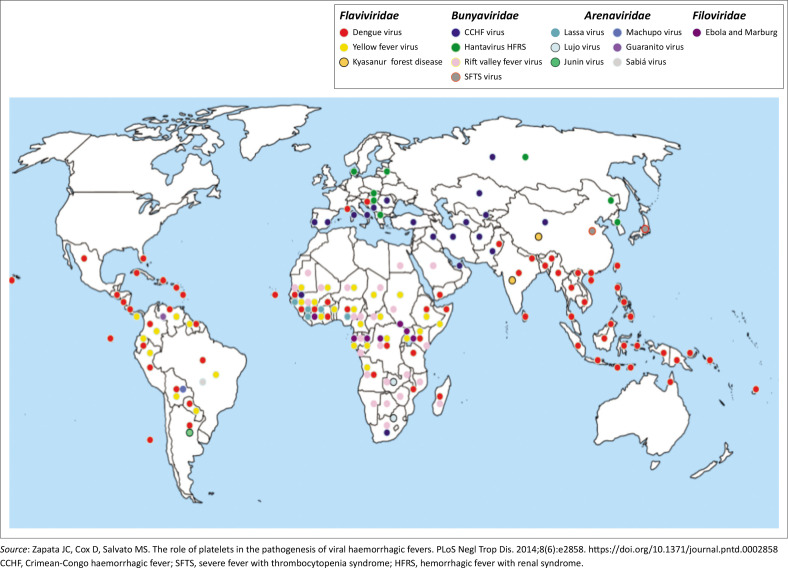
Geographical distribution of Viral haemorrhagic fevers globally.

Viral haemorrhagic fevers are prevalent in many parts of the world (Figure 1).^6^ The most common VHF in Southern Africa is the tick-borne Crimean-Congo haemorrhagic fever (CCHF).^3^ Rift Valley fever (RVF), a zoonotic disease of sheep and cattle, also causes human infections seen during major outbreaks in livestock, which occur when heavy rains facilitate the breeding of mosquitoes.

**FIGURE 2:**
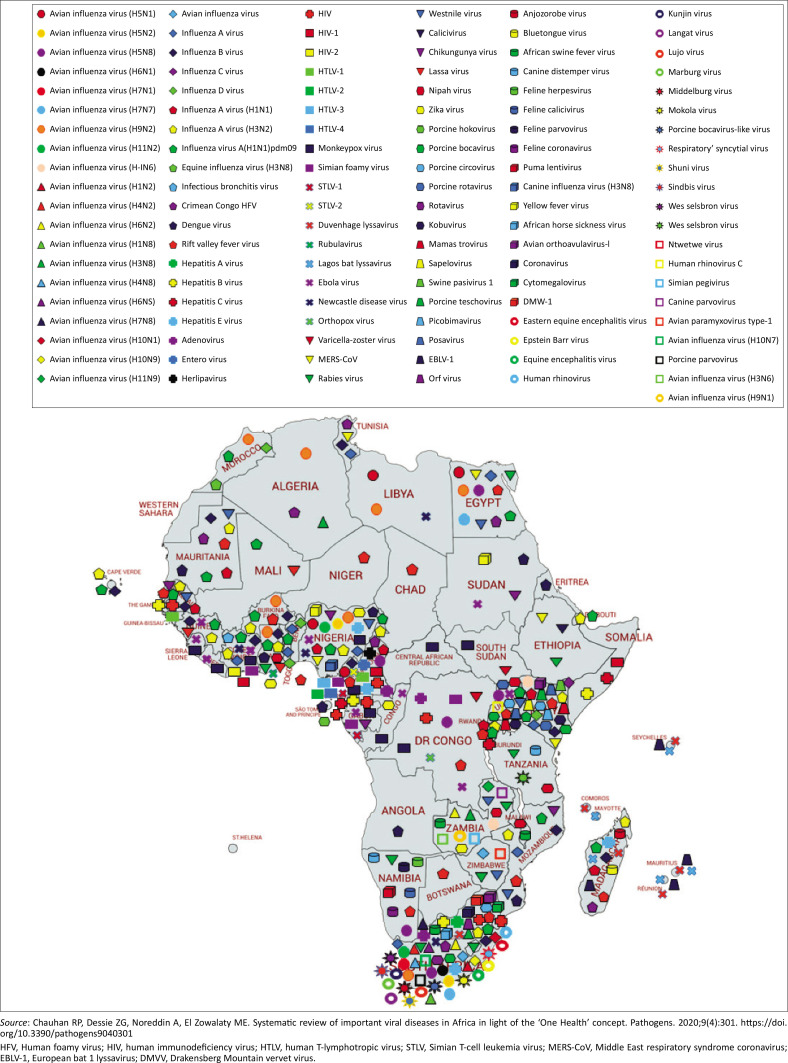
Map of Africa showing vector-borne and zoonotic virus diseases in Africa until September 2019.

There are increasing numbers of ill patients from countries in tropical Africa who seek medical attention in South Africa, and hence the risk of importing VHF to South Africa. Fatal VHFs, nosocomial infections, have occurred in South African hospitals.^3,7^ A recent systematic review of viral diseases has highlighted the trend of emerging and re-emerging virus diseases in Africa (Figure 2).^8^ In the light of the spread of the recent Severe Acute Respiratory Syndrome CoV-2 (SARS CoV-2) causing the COVID-19 disease, the awareness, diagnosis and management of zoonotic viral diseases becomes very significant.^8^

Viral haemorrhagic fevers should be considered in the differential diagnosis of every patient with unexplained fever, signs of haemorrhage and a history of travel within 21 days to an endemic area or history of contact.

Here follows a brief account of VHFs important for public health in South Africa, presented per virus genus.

### The Arenaviruses

Lassa and Lujo viruses are the two main arenaviruses from the family Arenaviridae that cause human diseases in Africa.^7^ Individuals can become infected with Lassa virus when they come into contact with the excrement or urine of an infected rodent through the ingestion of contaminated food or direct contact on broken skin.^2^

Lassa fever is generally a mild disease with fever and a death rate of 1% – 2%; however, in hospitalised patients, the death rates may approach 20% – 40%.^3,7,9^ The incubation period is 7–10 days. There is insidious onset of fever, chills, malaise, headache, generalised myalgia and prostration. Within 2–3 days, patients develop sore throat, vomiting, abdominal or retrosternal pains, cough, hypotension and bradycardia. Conjunctiva is injected, and there is lymphadenopathy, muscle tenderness, pulmonary rales and sometimes maculopapular rash. Progression to severe fever, toxaemia with haemorrhages, puffiness of the face and neck, hydrothorax, disorders of the central nervous system and shock occurs from day 5.^3,7,10^ Lassa fever is the most common cause of imported haemorrhagic fevers in non-endemic countries, and a high degree of suspicion coupled with clinical features and exposure history is important.^11^

Lujo mammarenavirus causes Lujo haemorrhagic fever (LUHF). It was named after the two cities involved in the 2008 outbreak, Lusaka in Zambia and Johannesburg in South Africa. During the outbreak, only five patients were diagnosed with LUHF, with an 80% case fatality rate.^2^ Amongst those patients diagnosed with LUHF, the symptoms resembled those of a severe form of Lassa Fever with an incubation period of 9–13 days. A prodromal illness, characterised by fever, headache and myalgia, followed by diarrhoea, pharyngitis, a morbilliform rash on the face and trunk may present on days 6–8 of illness without significant haemorrhage.^2,3^ All patients who were infected showed low blood platelets, low white blood cell counts and elevated liver function values on admission.^3^

Currently, there is no approved vaccine for arenaviruses in Africa. Treatment is limited to supportive therapy and antivirals like Ribavirin. Maintenance of appropriate fluid and electrolyte balance is crucial. Saturation level and blood pressure readings should be monitored, as well as treatment of any other complicating infections.^2^

### Bunyaviruses

Crimean-Congo haemorrhagic fever and RVF are caused by arthropod-borne bunyaviruses. These bunyaviruses are both reported from South Africa.

Crimean-Congo haemorrhagic fever is the most common haemorrhagic fever in South Africa caused by a tick-borne virus. The disease has a fatality rate of 30% – 50%.^2,5^ Humans acquire the infection from a bite of the *Hyalomma* tick or contact of broken skin with fresh infected blood and tissues of livestock. The livestock undergo only a benign infection.^3,9^

The incubation period commonly ranges from 1 to 3 days; however, it can be up to 12 days.^3,7^ Infection usually results from squashing ticks between the fingers. Onset is sudden, with severe headache, dizziness, neck pain and stiffness, photophobia, fever, chills, followed rapidly by myalgia with intense backache or leg pain, nausea, sore throat, non-localised abdominal pain, diarrhoea and vomiting.^3,7^ Fever is intermittent, and patients may undergo sharp changes of mood from days 1 to 2. By days 2–4, the patient presents with lassitude, depression and somnolence. A petechial rash appears on the trunk and limbs by days 3–6 of illness.^7,10^ Oozing of blood from injection or venepuncture sites, epistaxis, haematemesis, haematuria, melaena, gingival and vaginal bleeding or other orifices may commence on days 4–5 of illness. Severely ill patients enter a state of hepatorenal and pulmonary failure from about day 5 and progressively become drowsy, stuporous and comatose. Patients improve on days 9–10.^3,7^

During the second week, there is a decline of leucocytosis or even leucopoenia, and elevated aspartate aminotransferase (AST) and alanine transaminases (ALT), gamma-glutamyl transferase, lactic dehydrogenase, alkaline phosphatase and creatine kinase levels, whilst bilirubin, creatinine and urea levels increase.

Serum protein levels are also increased. During the first 5 days of illness, any of the following clinical pathology values are highly predictive of a fatal outcome: leucocyte counts ≥ 10 × 109/L; platelet counts ≤ 20 × 109/L; AST ≥ 200 U/L; ALT ≥ 150 U/L; activated partial thromboplastin time (APTT) ≥ 60 s; and fibrinogen ≤ 110 mg/dL.^3,7,12^ A scoring system for the diagnosis of CCHF is presented in Table 1.^13^ Supportive therapy and prevention are key. Ribavirin has been used successfully as the drug of choice in some cases.

**TABLE 1:** Criteria for clinical diagnosis of Crimean-Congo haemorrhagic fever.

Criteria	Incubation period following known or potential exposure	
	< 1 week	> 1 week undetermined
**History of exposure to infection**		
Bitten by tick(s) or crushed tick with bare hands	3	2†
Had direct contact with fresh blood or tissues of livestock	3‡	3†††
Had direct contact with blood, secretion or excretions of confirmed or suspected CCHF patient	3	2
**Signs and symptoms**		
Sudden onset	1	-
Fever > 38 °C on at least one occasion	1	-
Severe headache	1	-
Myalgia	1	-
Nausea and/or vomiting	1	-
Bleeding tendency: ecchymosis, epistaxis, haematemesis, haematuria or melaena	3	-
**Clinical pathology during first 5 days of illness:**		
Leukopenia or leucocytosis	-	-
WCC < 3 × 109/1 or > 9 × 109/1	1	-
Thrombocytopenia	-	-
Platelets < 150 × 109/1	1	-
Platelets < 100 × 10/1	2	-
> 50% decrease in either WCC or platelet count within 3 days	1	-
Abnormal PI	1	-
Abnormal PTT	1	-
Raised transaminases	-	-
AST > 100 u/L	1	-
ALT > 100 u/L	1	-

*Source*: Swanepoel R. Recognition and management of viral haemorrhagic fevers: A handbook and resource directory [homepage on the Internet]. 2nd ed. Sandringham: National Institute for Virology, Department of Health, South Africa. 1987 [cited 2020 Mar 30]. Available from: https://www.kznhealth.gov.za/medicine/viral.pdf^13^

Note: A score of ≥ 12 points constitute an indication for treating a patient as a case of CCHF.

PI, Prothrombin Index; PTT, Partial Thromboplastin Time; WCC, White Cell Count; CCHF, Crimean-Congo haemorrhagic fever; AST, aspartate aminotransferase; ALT, alanine transaminases.

† , South African tick-borne and ehrlichiosis must be excluded.

‡ , Rift Valley Fever must be excluded.

††† , Brucellosis, Q Fever and anthrax must be excluded.

Rift Valley fever is a mosquito-borne virus disease of livestock found in Africa and Madagascar, with some outbreaks reported in Saudi Arabia and Yemen,^2^ which affects sheep and cattle. It is caused by a mosquito-borne virus. Most patients experience benign illness with fever, some with ocular sequelae and only < 1% develop fatal haemorrhagic disease, hepatitis or encephalitis. However, massive outbreaks have occurred resulting in large numbers of human deaths.^3^

The incubation period is 2–6 days, and infections are mild, or moderate to severe febrile illness, with sudden onset of severe retro-orbital pain and headache, photophobia, suffused conjunctivae, myalgia, arthralgia, nausea and tenderness of the liver without hepatomegaly (Table 2).^2,7,9,10^ The disease may run a diphasic course over 2 weeks (3 days of fever, 1–2 days of remission, followed by another 2–3 days of fever).^9^ Ocular complications occur in 5% – 20% of cases, 1–3 weeks after the onset of illness. Vision improves over a period of 1–3 months, as lesions resolve. Retinal detachment and blindness can occur. Less than 0.5% of patients develop encephalitis or haemorrhagic disease with high death rates.^3^ No vaccine is currently available.^2^ Effective preventive measures should be instituted to avoid early exposure to blood or tissues of animals that may potentially be infected.^2^

**TABLE 2:** Characteristics viral haemorrhagic fever in Africa.

Virus	Geographical distribution	Presenting symptoms	Timing from symptoms to rash	Exanthem features	Patient with exanthema
Rift Valley fever	South eastern/western/northern Africa, Madagascar, Yemen	Fever, myalgia, dizziness, headache, mood swings, tachycardia	2–4 days	Petechial rash and/or a petechial enanthem, involving mouth and/or throat	1%
Lassa virus (rodent associated)	West Africa	Fever, chills, malaise, swollen face and neck, sore throat	No rash Incubation period, 7–10 days	Sometimes maculopapular rash on the trunk	Not a significant finding
Lujo virus (rodent associated)	Africa-Zambia	Fever, headache sore throat, myalgia	9–13 days	Morbilliform rash on the face and trunk	Not a significant finding
Hanta viruses (rodent associated)	Europe, Asia North and South America	Depends on the clinical staging	Incubation period, 2–3 weeks	Butterfly rash or no rash	Not a significant finding

*Source*: Centers for Disease Control and Prevention. Viral Haemorrhagic fevers [homepage on the Internet]. [cited 2020 Apr 13]. Available from: https://www.cdc.gov/vhf/index.html

### Filoviruses

Ebola and Marburg viruses are classified as filoviruses and can cause severe or fatal haemorrhagic fever in primates that are both human as well as non-human. Filoviruses are therefore characterised as zoonotic and originate from fruit bats in Africa.^5^ Person-to-person transmission is the means by which further transmission occurs. Direct contact with infected bodily fluids, such as blood, faeces or vomitus, can lead to transmission.^2,5^ The signs and symptoms of Ebola and Marburg virus diseases are like those of the other VHFs.^5^

Ebola virus disease (EVD) and Marburg virus disease (MVD) have similar clinical features presenting in three phases: initially fever, headache and myalgia; followed by gastrointestinal symptoms, including diarrhoea and vomiting; and then dehydration. In the second week, there may be recovery or deterioration with collapse, neurological manifestations and unexplained haemorrhaging, bleeding and bruising that can lead to a fatal outcome.^2,5^

The incubation period is from 2 to 21 days. Patients present with a sudden onset of fever, severe headache, sore throat, chest and/or abdominal pain, myalgia, fatigue, nausea and anorexia. Signs include oral/throat lesions, persistent diarrhoea and vomiting, dehydration, dry cough, conjunctivitis and non-itching maculopapular rash of trunk and limbs, with onset about day 5 of illness and desquamation 4–10 days later. The more severe and fatal cases progress to a haemorrhagic state by days 5–8.^3,5^

The primary health care practitioner should know and understand the case definition for EVD and person under investigation (PUI) according to the National Institute for Communicable Diseases (NICD).^2^

If a diagnosis of EVD is suspected, the patient should be isolated immediately in a single room, with a private bathroom. The personnel should use appropriate personal protective equipment (PPE).^2^ The infection control personnel should be informed immediately.^2^ Ebola haemorrhagic fever has become one of the world’s most feared pathogens and in July 2019 was declared a public health emergency of international concern.^14^

## Diagnosis

### Clinical diagnosis of viral haemorrhagic fever

Non-specific early signs include fever, headache, pharyngitis, myalgia, vomiting, abdominal pain and diarrhoea. Viral haemorrhagic fevers are easier to diagnose. Once petechial rash or ecchymoses develops, other haemorrhagic signs, such as epistaxis, haematemesis and melaena, become apparent.^7^ There is a rapid progression to jaundice, shock, altered mental state and multi-organ failure.^3,9^

### Features that support a diagnosis of viral haemorrhagic fever

- Short duration and rapid progression of disease.
- Lack of evidence in the patient’s history or physical examination to exclude VHF.
- Leucopenia, thrombocytopenia, coagulation abnormalities and raised serum transaminases.
- The progression of the illness and the timing of bleeding in relation to the onset of symptoms are important in guiding the diagnosis of VHF versus alternative diagnosis.

### Procedure to follow when viral haemorrhagic fever is suspected

Laboratory personnel should be warned of the suspected diagnosis. It should be ensured that all new and old specimens have been submitted to the NICD in Johannesburg, South Africa, for specific VHF diagnostic tests. Specimens from live patients should include 5 mL – 10 mL of clotted blood, 5 mL of blood taken with Ethylenediaminetetraacetic acid (EDTA) and a throat swap in viral medium.^3^

### Immediate action to be taken after clinical diagnosis of viral haemorrhagic fever

- Inform the management of the suspected case.
- Isolate the patient and apply infection precautions.
- Initial management is supportive, for example, blood/fluid therapy and verify the diagnosis.
- Identify and inform contacts, for example, staff members who must be placed under observation.
- Notify the local and provincial communicable disease control coordinator.
- Inform the NICD hotline (0828839920).
- Manage the patient appropriately using supportive therapy, including fluid management.
- If VHF is suspected, transfer the patient to a hospital suited to managing the case.
- Assess the status of the patient as either low, moderate or high risk with respect to VHF.^3,13^

### Low-risk patients

Febrile disease with features suggestive of VHF but are not severely ill and lack a history of contact with known VHF patients or animals, or animal tissues or ticks and mosquitoes, and have no history of travel 3 weeks prior to the onset of illness.

### Moderate-risk patients

Febrile disease with features suggestive of VHF, and are not severely ill, but have visited or resided in a tropical or rural environment, or have had indirect contact with animals, animal tissues, ticks and mosquitoes or VHF patients during the 3 weeks preceding the onset of illness.

### High-risk patients

Severely ill, with fever and haemorrhagic manifestations, may be associated with a history of travel 3 weeks prior to development of symptoms or definite exposure to VHF. Hospital and laboratory staff or relatives who had contact with a confirmed VHF patient and developed symptoms within 3 weeks are included.^3,9^

### Management of viral haemorrhagic fever patients

#### Antiviral therapy

Ribavirin is a synthetic nucleoside analogue, which is useful in treating arenavirus infections. The drug is used ‘off-label’ for the treatment of CCHF or Lassa fever (Table 3).^4,9^ Ideally, all severely ill patients should be treated with the intravenous formulation of Ribavirin, which is currently unavailable in South Africa.^3,7,12^ Management at a primary health care facility is hence supportive, whilst maintaining haemodynamic stability prior to referral to higher levels of care.

**TABLE 3:** Therapeutic and preventative measures specific for selected viral haemorrhagic fever.

Arenaviruses	Treatment	Prevention
Lassa fever	Supportive, ribavirin	Rodent control, avoidance of reservoir
Filoviruses	-	-
Ebola HF	Supportive	Ervebo vaccine
Marburg HF	Supportive	Unknown
Bunyaviruses	-	-
Crimean-Congo HF	Supportive, ribavirin (controversial)	Tick control, avoidance of slaughtered animals
Rift Valley fever	Supportive	Avoidance of slaughtered animals

*Source*: Ericsson CD, Steffen R, Isaäcson M. Viral Hemorrhagic fever hazards for travelers in Africa. Clin Infect Dis. 2001;33(10):1707–1712. https://doi.org/10.1086/322620

HF, haemorrhagic fever.

## Conclusion

A haemorrhagic state may be caused by many different infective conditions and not all are fatal. It is usually as a result of intense circulation of microorganisms that also cause liver damage. Systematic consideration of all possible causes, including a detailed travel history, and appropriate laboratory investigations, usually provides the diagnosis. Suspected infections must be treated with strict infection control measures, and contacts must be identified, traced and isolated. Primary health care workers must have a high index of awareness and suspicion, and follow guidelines whenever they are confronted with suspected cases. All primary health care facilities must be equipped with ready-to-use PPEs.
